# Notes on the systematics of Cuscutasect.Subulatae (subg. Grammica) with the description of *Cuscutamantiqueirana*, a new species from Brazil

**DOI:** 10.3897/phytokeys.184.69037

**Published:** 2021-10-26

**Authors:** Mihai Costea, Simone Soares da Silva, Rosângela Simão-Bianchini, Ana Rita G. Simões, Saša Stefanović

**Affiliations:** 1 Department of Biology, Wilfrid Laurier University, Waterloo, Ontario N2L 3C5, Canada Wilfrid Laurier University Waterloo Canada; 2 Núcleo de Pesquisa Curadoria do Herbário, Instituto de Botânica, Avenida Miguel Stéfano, 3687, Vila Água Funda, São Paulo, Brazil Núcleo de Pesquisa Curadoria do Herbário, Instituto de Botânica Sao Paulo Brazil; 3 Royal Botanic Gardens, Kew, Richmond, Surrey TW9 3AE, UK Royal Botanic Gardens Kew Richmond United Kingdom; 4 Department of Biology, University of Toronto, Mississauga, Ontario L5L 1C6, Canada University of Toronto Toronto Canada

**Keywords:** Convolvulaceae, dodders, ITS, morphology, parasitic plant, phylogeny, systematics

## Abstract

*Cuscutamantiqueirana* Costea, S.S. Silva & Sim.-Bianch. a new species from montane cloud forests of the Serra da Mantiqueira, Brazil, is described and illustrated. The morphological and phylogenetic analyses revealed that the new species belongs to sect. Subulataeofsubg.Grammica. The new species is related to C.odoratavar.botryoides, *C.rotundiflora* and *C.globiflora* from which it differs in narrower calyx lobes and the presence of four stomatiferous lobes or projections at the distal part of the ovary. A detailed morphological comparison with C.odoratavar.botryoides, morphologically the most similar taxon, is provided along with the geographical distribution, ecology and host range of the species. The morphological and phylogenetic relationships of the new species, as well as the diversity of stomatiferous projections, are discussed in the broader context of sect. Subulatae and subg. Grammica. Cuscutabolivianavar.paranensis is considered a synonym of C.odoratavar.botryoides.

## Introduction

With ca. 30 species, Cuscutasect.Subulatae (Engelm.) Costea & Stefanović is the largest infrageneric group of subg. Grammica (Lour.) Peter, and of *Cuscuta* L. in general (Costea et al. 2015). The section was recently circumscribed (Costea et al. 2015) based on a core of species included by Engelmann (1859) in subsect. Subulatae. This infrageneric clade has diversified in South America (Yuncker 1922, 1932; Stefanović et al. 2007; García et al. 2014), but it also contains two African species, *C.kilimanjari* Oliv. (distributed across Tropical East Africa, Central Africa and Madagascar) and *C.blepharolepis* Welw. ex Hiern. (a more enigmatic taxon known only from two collections in Guinea and Angola, in Western Africa). Although *C.blepharolepis* has not yet been sampled, *C.kilimanjari* was found to be nested deeply within this clade, as sister to *C.cristata* Engelm. (S. Brazil to N. Argentina), strongly suggesting long-distance dispersal (Stefanović et al. 2007; García et al. 2014). Flowers of sect. Subulatae are among the largest in *Cuscuta*, often fleshy, and in many species apparently cross-pollinated (Wright et al. 2012). Infrastaminal scales, which are unique structures with defence role in *Cuscuta* flowers (Riviere et al 2013), are very diverse in shape, size and number of fimbriae; in a few species they are entirely reduced while in others they possess densely papillate fimbriae (Riviere et al. 2013). Pollen is also more varied among species than in other sections of subg. Grammica; it can be 3, 4, 5 or 6–7-colpate, and tectum can be imperforate, perforate, microreticulate, or reticulate (Welsh et al. 2010). Styles are thick, cylindrical or subulate, and stigmas are large, convoluted and lobed (Yuncker 1932; Wright et al. 2011). The fruit is usually dehiscent (Ho and Costea 2018) with large seeds (Olszewski et al. 2020). Plastome evolution studies have revealed extensive losses of plastid genes, including the otherwise highly conserved small and large ribosomal subunits (Braukmann et al. 2013).

Engelmann (1859), Yuncker (1921, 1922, 1923, 1932) and Hunziker (1947, 1949, 1950) described the majority of taxa in sect. Subulatae, but this clade has not been revised at species level to date. Among the practical obstacles towards a taxonomic revision of this section are the scarcity of herbarium material available and notoriously difficult DNA extraction from herbarium specimens. After plants dry, they often become brown-blackish and more difficult to analyze morphologically. Plastid sequences, which have been used extensively to reconstruct phylogeny of the entire genus (García et al. 2014),subgenus Grammica (Stefanović et al. 2007), and multiple clades of the latter subgenus (e.g., Costea et al. 2008, 2011a, 2011b, 2013, 2020; Costea and Stefanović 2009), cannot be employed for this section because of the plastome reductions (Braukmann et al. 2013).

The objective of this study is to report a new species in sect. Subulatae, as well as to discuss its putative relationships with other taxa in this group. The new species has been discovered independently both among herbarium specimens and by doing field work.

## Materials and methods

*Cuscuta* specimens from the following herbaria were examined and annotated: AAU, B, BAB, BM, BR, BRIT, CAS, CEN, CORD, CTES, DAO, DIAM, E, ESA, F, G, GH, HB, HRCB, HUEFS, HUFU, HUSC, IAC, JEPS, K, L, LIL, LP, LPB, LPS, MA, MBM, MEL, MERL, MEXU, MICH, MO, NY, OXF, P, PACA, PMSP, QCNE, R, RB, RSA, S, SGO, SI, SJRP, SP, SPF, SPSF, TEX, TRTE, UB, UCR, UEC, UPCB, UPRRP, UPS, US, W, and WLU (Herbaria acronyms from Thiers 2018-continuously updated). In addition, we conducted a series of targeted fieldtrips to Serra da Mantiqueira, which included Itatiaia National Park (Rio de Janeiro), São José dos Campos, São Francisco Xavier, Pindamonhangaba, Campos do Jordão (São Paulo) and Camanducaia (Minas Gerais) to observe the species in the field and collect additional samples for the molecular analysis. We also provisionally assessed the conservation status of the new species using georeferenced herbarium specimens mapped in GeoCAT (Bachman et al. 2011). This rapid geospatial analysis tool determines the extent of occurrence (EOO) and the area of occupancy (AOO) and assigns a conservation status based on criteria B1 and B2 established by IUCN Standards and Petitions Subcommittee (2019). The GeoCAT file is available at: https://1drv.ms/u/s!Aj8HnxOfICaFgpo6hJhTTuzBNhFV2w?e=BLBf0i.

### Molecular phylogenetic analyses

Of the 28 collections of individuals belonging to the new species *C.mantiqueirana*, three specimens (Appendix 1) were found to be of sufficient quality and quantity for molecular studies. To infer the phylogenetic affinities of this species within Cuscutasect.Subulatae, we obtained sequences from the internal transcribed spacer (ITS) region of nuclear ribosomal DNA (rDNA). DNA extractions, polymerase chain reaction (PCR) reagents and conditions, amplicon purifications, cloning, and sequencing procedures follow Stefanović et al. (2007) and Stefanović and Costea (2008). The sequences generated in this study have been submitted to GenBank (accession numbers MZ389688–MZ389691). Using Se-Al v.2.0a11 (Rambaut 2002), newly obtained sequences were incorporated into previously aligned nrITS matrix of accessions from Cuscutasect.Subulatae (Stefanović et al. 2007; Stefanović and Costea 2008; deposited in TreeBASE under study number S1929). Based on these, more inclusive analyses, we selected *C.microstyla* Engelm. as a functional outgroup.

Phylogenetic analyses were conducted under parsimony and maximum likelihood using PAUP* v4.0b10 (Swofford 2002). Sequence data were treated as unordered and all changes were equally weighted. Gaps in the alignments were treated as missing data. Given the moderate number of terminal units, the parsimony searches were conducted with a Branch-and-Bound algorithm, ensuring recovery of all of the most parsimonious (MP) trees. The full heuristic searches for maximum likelihood (ML) trees were performed under the general time-reversible (GTR) model of DNA substitution (Lanave et al. 1984), with the rate of variation among nucleotides following a discrete gamma distribution and allowing for invariable sites (GTR+G+I), involving 100 replicates with stepwise random taxon addition, tree bisection-reconnection (TBR) branch swapping, and MULTREES option on. All model parameters were estimated from data, except the base composition, where empirical frequencies have been used. The support for clades was inferred by nonparametric bootstrapping (Felsenstein 1985), under parsimony, using 1,000 heuristic bootstrap pseudoreplicates, TBR branch swapping, and MULTREES option on. Support for a relationship was considered weak if bootstrap value was < 70%, moderate if between 70 and 90%, and strong if > 90%.

### Microscopy

Flowers, fruits and seeds removed from herbarium specimens were steeped in gradually warmed 50% ethanol, which was then allowed to boil for a few seconds to rehydrate tissues. An ethanol solution is more suitable for rehydration than simple water because it removes some of the dark pigments that result after drying, and at the same time, it hardens the tissues, which are very delicate in the *Cuscuta* flowers. For basic morphology, flowers were dissected under a Nikon SMZ 1500 stereomicroscope and imaged with PaxCam Arc digital camera equipped with a PAX-IT 8.2 (MIS Inc. 2021, Villa Park, Illinois) imaging software. Numerous photographs illustrating details of the floral and fruit morphology for all taxa, including their type collections, are made available on the Digital Atlas of *Cuscuta* website (Costea 2007-onwards). To examine finer (micro)morphological features, rehydrated flowers, fruits and seed samples were dehydrated through an ethanol series (50%, 70%, 85%, 95%, and 100%; each step 1h) and then critically point dried with Tousimis Autosamdri-931. Samples were mounted on aluminum stubs and sputter-coated with 30 nm of gold using Emitech K550 sputter coater. Examination, imaging and measurements were made using a Hitachi SU1510 scanning electron microscope (SEM) at 5–10 kV.

## Results

### Molecular phylogeny

**Figure 1.**
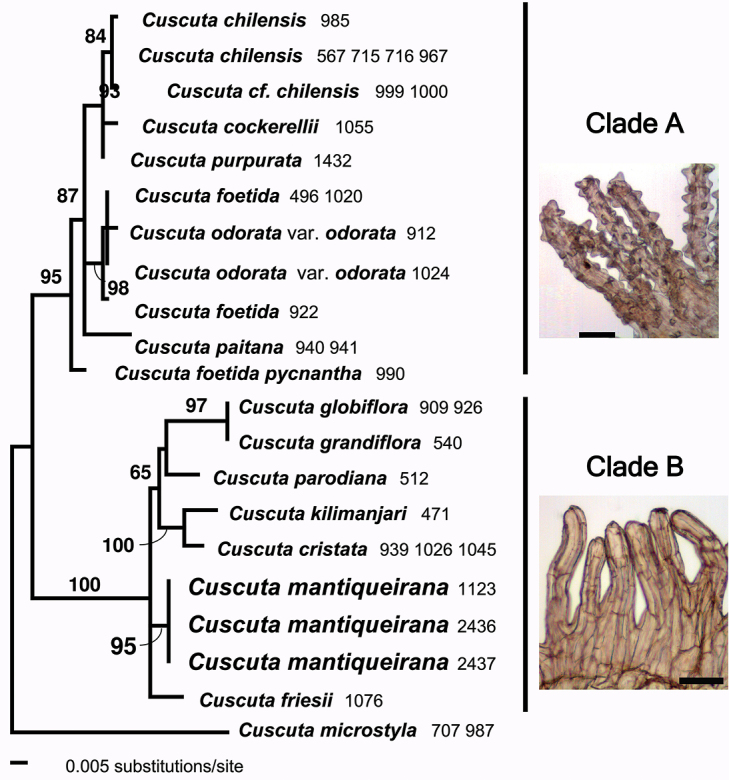
Phylogenetic relationships among species of Cuscutasect.Subulatae shown as a phylogram obtained from maximum likelihood analysis of nrITS sequence data under the GTA+G+I model of DNA evolution. Tree is rooted using *C.microstyla* as a functional outgroup. Numbers following species names correspond to DNA accessions (Appendix 1). Bootstrap values are indicated. Photos represent fimbriae of infrastaminal scales: papillate in clade A and not papillate in clade B. Scale bars: 100 μm.

The parsimony analysis resulted in 12 MP trees [length = 161; consistency index (CI) = 0.907; retention index (RI) = 0.957]. The maximum likelihood analysis resulted in a single ML tree, topologically fully compatible with the strict consensus of the MP trees. The ML phylogeny was selected to illustrate the inferred relationships in this section, including the placement of *C.mantiqueirana*, as well as branch lengths (Fig. 1). Based on the strong support, as measured by bootstrap values, and the sequence divergence, as indicated by the branch lengths, molecular data revealed three major lineages within Cuscutasect.Subulatae (Fig. 1). These results are fully consistent with our previous findings (Stefanović et al. 2007; Stefanović and Costea 2008).

*Cuscutamicrostyla* formed a distinct lineage within sect. Subulatae (Fig. 1). This Chilean-Argentinean species is restricted to high Andes, and represents an exception in the section in its relatively small flowers and indehiscent fruits. The second lineage (Clade A; 95% BS; Fig. 1) consisted of species (e.g., *C.chilensis* Ker Gawl., *C.cockerellii* Yunck., *C.foetida* Kunth, C.odorataRuiz & Pav.var.odorata, *C.paitana* Yunck., *C.purpurata* Yunck.; Fig. 1) with tubular-campanulate flowers, papillate infrastaminal scales (Riviere et al. 2013), and 3-colpate pollen grains (Welsh et al. 2010) primarily distributed along the Andes (Ecuador, Peru, Bolivia, Chile). Finally, the third lineage (clade B; 100% BS; Fig. 1) that also included *C.mantiqueirana*, is characterized by flowers with rotate or globose to urceolate corollas, infrastaminal scales without or with only a few papillae (Riviere et al. 2013), and usually (although some exceptions are known) 4–6-colpate pollen grains (Welsh et al. 2010). This lineage includes *C.cristata* Engelm., *C.globiflora* Engelm., *C.friesii* Yunck., *C.grandiflora* Kunth, *C.kilimanjari*, *C.parodiana* Yunck. (Fig. 1; clade B), and most likely, based on their morphological similarity, several other species that could not be included in the molecular analysis: C.odorataRuiz & Pav.var.botryoides Engelm., *C.rotundiflora* Hunz., and *C.boliviana* Yunck. Members of this morphologically identified clade occur east of the Andes in Argentina, Uruguay and Brazil, but a few are found along the Andes (Colombia to Chile). An identification key for the taxa of clade B is included below. Among these taxa, *C.mantiqueirana* is morphologically most similar, and geographically closest to C.odoratavar.botryoides, and a comparison between the two taxa is provided in Table 1. Based on our current sampling, *C.mantiqueirana* is reciprocally monophyletic and molecularly distinct from other members of this clade, as evidenced by the branch length subtending it and strong bootstrap support (Fig. 1). The molecular results agree with the morphological distinctiveness of all the species considered, suggesting that taxa for which DNA could not be extracted, but which are morphologically distinct, will also be validated as discrete lineages when molecular data become available.

### Identification key for taxa within clade B (see Fig. 1) of section Subulatae

**Table:** 

1	Infrastaminal scales with densely papillate fimbriae	**(Clade A)**
–	Infrastaminal scales without papillae on fimbriae or only with a few distal papillae (scales sometimes absent in *C.kilimanjari*)	**2 (Clade B)**
2	Corolla tubular-cylindrical becomes tubular-urceolate at fructification	***C.parodiana***
–	Corolla rotate or campanulate becomes globose to urceolate-globose at fructification	**3**
3	Corolla membranous, rotate	***C.friesii* , *C.argentinana***
–	Corolla fleshy, campanulate becomes globose to urceolate-globose at fructification	**4**
4	Fruit indehiscent to late irregularly-dehiscent	***C.cristata***
–	Fruit circumscissile dehiscent	**5**
5	Infrastaminal scales absent or with a few fimbriae; Africa	***C.kilimanjari***
–	Infrastaminal scales well developed with numerous fimbriae; South America	**6**
6	Corolla lobes erect-connivent; stigmas conical	***C.globiflora***
–	Corolla lobes spreading or reflexed; stigmas globose or depressed	**7**
7	Stamen filaments and styles evidently subulate; stigmas 1–1.5 mm wide	***C.rotundiflora***
–	Stamen filaments and styles cylindrical or only slightly subulate; stigmas 0.3–0.7 mm wide	**8**
8	Pedicels obconical; flowers (4–) 5–7 mm long; external calyx lobes usually not carinate; capsule with a collar around the interstylar aperture	***C.mantiqueirana***
–	Pedicels cylindrical; flowers 3.8–5 mm long; external calyx lobes carinate; capsule without a collar around the interstylar aperture	**C.odoratavar.botryoides (= C.bolivianavar.paranensis)**


### Taxonomic treatment

#### Cuscuta mantiqueirana

Taxon classificationPlantaeSolanalesConvolvulaceae

Costea, S.S.Silva, Sim.-Bianch. sp. nov.

7CA4ABB3-1FC0-542D-BA9F-906405F3F8D1

urn:lsid:ipni.org:names:77221396-1

**Figure 2.**
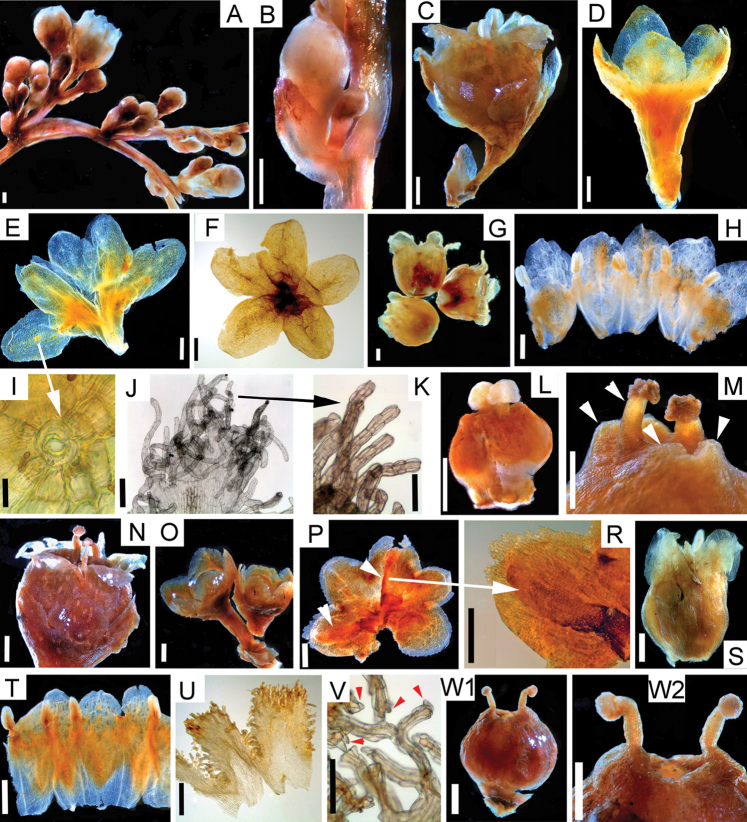
Comparative morphology of *Cuscutamantiqueirana* and C.odoratavar.botryoides using rehydrated herbarium material **A–M***Cuscutamantiqueirana***A** inflorescence **B** incipient stage of inflorescence development showing bract and developing flowers **C** flower and bract **D** calyx 3D, note the receptacle prolonging into obconical pedicel **E, F** calyx dissected, variation **G** corolla 3D **H** corolla dissected to show infrastaminal scales and stamens **I** stoma on calyx lobe, note that large protuberances with stomata do not develop in this species **J** distal part of infrastaminal scale **K** detail of infrastaminal scale fimbriae, note that no papillae are present **L** gynoecium **M** detail of distal part of ovary showing 4 lobes with stomata flanking the two styles. All images were obtained from *G.F. Árbocz* et al. *2750* except A, B, H and L which are from the type, *Simão-Bianchini 1241***N–W**Cuscutaodoratavar.botryoides**N** flower **O** calyces 3D **P** calyx dissected, note the broader calyx lobes, two of them with prominent carinas that bear stomata (indicated by arrows) **R** detail of carinate calyx lobe **S** corolla 3D **T** fragment of dissected corolla **U** infrastaminal scales **V** detail of fimbriae showing 1–2 papillae at their tips (arrows) **W1** gynoecium **W2** detail of distal part of ovary, note the absence of lobes with stomata. All images from *Lobb 49* except P, R = *Hatschbach 22109* and V = *Hoehne s.n.* Scale bars: 1 mm (**A–H, L, N–Q, S–U, W1, W2**); 50 μm (**I**); 0.5 mm (**J, M, R, K**); 0.25 mm (**V**).

**Figure 3.**
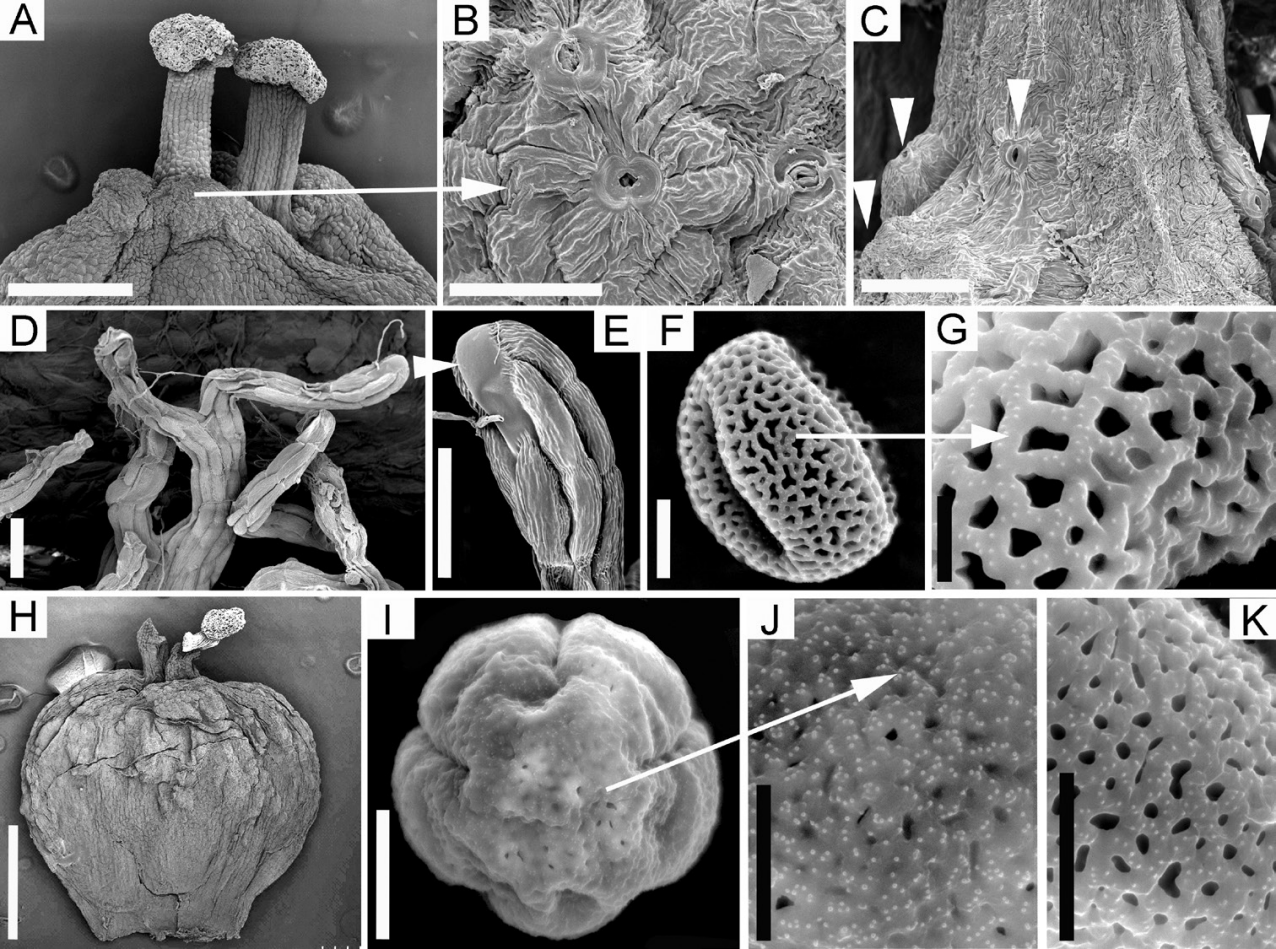
Micromorphology of *Cuscutamantiqueirana* and C.odoratavar.botryoides**A–G***Cuscutamantiqueirana***A** distal part of ovary **B** stomata on lobes flanking styles **C** stomata (indicated by arrows) at the base of styles **D–E** infrastaminal scale fimbriae and detail **F–G** 3-colpate, reticulate pollen. All images from *G.F. Árbocz* et al. *2750* except C which is from *Simão-Bianchini 1241***H–K**Cuscutaodoratavar.botryoides**H** gynoecium **I–K** pollen grain: 5-colpate with perforate tectum. All images from *Burkhart* 1626. Scale bars: 0.5 mm (**A**); 100 μm (**B–D**); 50 μm (**E**); 10 μm (**F, I**); 1 mm (**H**); 5 μm (**G, J, K**).

**Figure 4.**
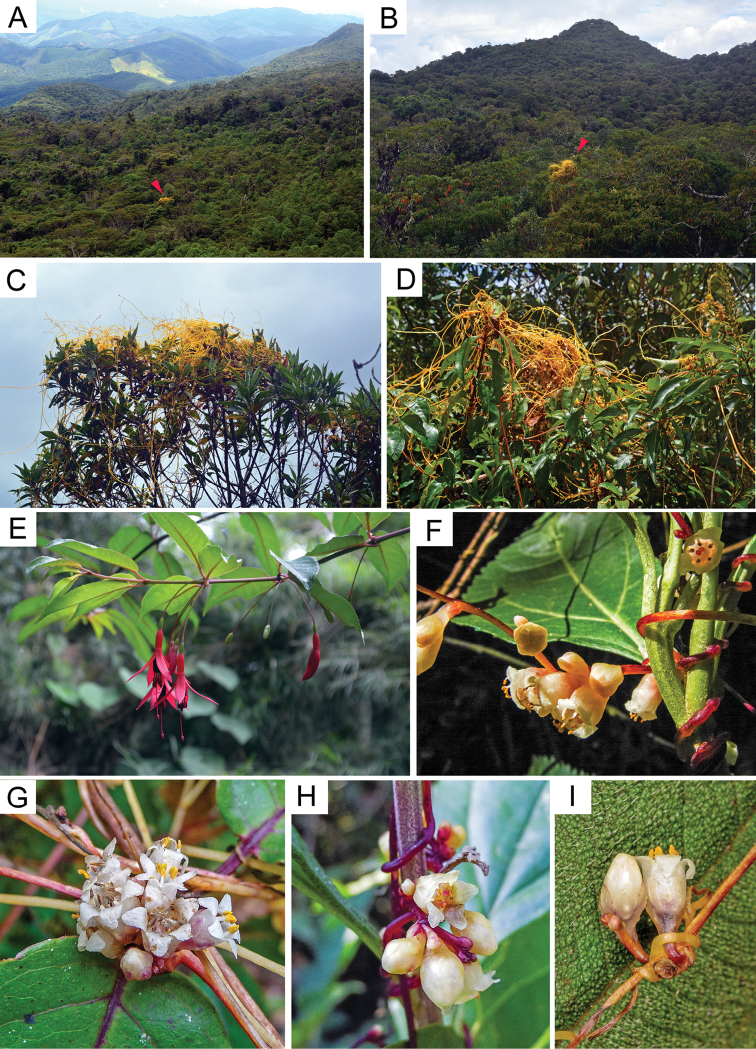
Habitat, habit, inflorescences and flowers of living *Cuscutamantiqueirana***A, B** montane cloud forest habitat (arrows indicate *C.mantiqueirana*) **C, D** habit **E** the most common host, *Fuchsiaregia***F–H** inflorescences **I** flowers (**F–I** photos: Suzana Ehlin Martins **F, H** Itatiaia **G, I** Serra do Papagaio).

Figs 2 , 3 , 4 ; Table 1

##### Diagnosis.

*Cuscutamantiqueirana* is similar to C.odoratavar.botryoides, *C.rotundiflora* and *C.globiflora* because of their corolla that becomes globose at fructification and their well-developed scales with numerous fimbriae, but it differs from all these taxa in narrower calyx lobes, the presence of four stomatiferous lobes or projections, 0.4–0.6 mm long at the ovary apex, which form a collar at the fruit stage, as well as 3-colpate pollen grains with reticulate tectum. It differs from C.odoratavar.botryoides in the obconical pedicels and larger flowers, 4.5–7 mm long (pedicels are cylindrical and flowers 5.5–5 mm long in C.odoratavar.botryoides). From *C.rotundiflora* it differs in the infrastaminal scales equalling or exceeding the corolla tube, cylindrical stamen filaments and styles, as well as stigmas 0.5–0.6 mm wide (in *C.rotundiflora* the scales are shorter than corolla tube, filaments and styles are subulate and stigmas 1–1.5 mm wide. From *C.globiflora* it differs in the spreading to reflexed corolla lobes and globose-depressed stigmas (in *C.globiflora*, corolla lobes are erect-connivent and stigmas conical).

##### Type.

Brazil. Minas Gerais: Camanducaia, Monte Verde. Parasita com ramos desde alaranjados até vináceos, sempre em local sombreado e humido. Flores amarelas, 22 IV 1999, *R.Simão-Bianchini 1241* (holotype: SP!; isotypes: K!, NY!, MBM!, R!, SPF!, UB n.v., UEC!, WLU!)

##### Description.

***Stems*** medium to coarse, yellow-orange, purple-tinged or entirely purple. ***Inflorescences*** paniculiform-racemiform, occasionally glomerulate, usually not confluent; ***pedicels*** 1.5–4 mm, thick, gradually tapering into the fleshy, conical receptacle; ***bracts*** 1 at the base of cymes, 1.1–2.5 mm long and 0.8–1.6 mm wide, fleshy, triangular-ovate, rounded, not carinate, margins entire. ***Flowers*** 5-merous, (4–) 5–7 mm long, fleshy, white when fresh, dark-brown when dried; papillae absent; ***laticifers*** ± visible, translucent in the calyx, corolla lobes, and more noticeable, dark-colored in the 1/3 distal half of the ovary; isolated, oblong-ovoid. A few stomata are present along the calyx mid-vein lobes, but stomatiferous carinas are usually absent; ***calyx*** 2.8–4 mm long, creamy-white to purple-tinged when fresh, dark-brown when dried, not reticulate, dull, cupulate, ca. 3/4 as long as corolla tube, divided 2/3–4/5 to the base, tube 0.5–1.5 mm long, lobes 2–2.5 mm long, ovate-oblong, longer than wide to as long as wide, the two external ones overlapping, usually not carinate, margins membranous, finely erose, not auriculate at base, apex rounded; ***corolla*** (4–) 4.5–7 mm long, tube 2.1–4.6 mm long, campanulate but becoming ± globose at fructification; lobes 1.8–2.5 mm long, initially erect, later reflexed, shorter than the tube, broadly ovate, overlapping, ± auriculate at base, margin entire to irregularly crenulate, apex rounded, straight; ***stamens*** exserted, shorter than the corolla lobes, anthers 0.8–1.2 mm long, elliptic to oblong, filaments 0.6–0.8 mm long; ***pollen*** 3-zonocolpate,19–29.5 μm long and 17–20 μm wide, subprolate to spheroidal or suboblate, tectum microreticulate to reticulate, lumina 0.9–3.3 μm in diameter; ***infrastaminal scales*** 3–5 mm long, equalling to slightly longer than corolla tube, ovate to oblong, bridged at 0.7–1.6 mm, densely fimbriatae, fimbriae 50–80, 0.5–1.2 mm long, thin-filiform without papillae distally; ***ovary*** apex on both sides of styles raised to form two pairs of lobes or projections with stomata, 0.4–0.6 mm long; ***styles*** 0.3–0.9 mm long, 0.3–0.5 mm thick, much shorter than the ovary, cylindrical to slightly subulate, sometimes also with stomata at their base; ***stigma*** 0.35–0.6 mm long and 0.5–0.7 mm wide, white to purple in the fresh flowers, globose to wider than long, convoluted and lobed. ***Capsules*** circumscissile, 4–4.8 mm long and 4–5.5 wide mm, globose to ovoid, with a collar around the large interstylar aperture, opaque, entirely surrounded by the withered, persistent corolla. ***Seeds*** 2 per capsule, 1.5–2.8 mm long and 2–2.4 mm wide, dorsiventrally compressed, subrotund, hilum area lateral, 0.7–1 mm in diameter, scar 0.25–0.30 mm long, seed coat alveolate/papillate. ***Chromosome number*** not known.

**Table 1.** Morphological comparison between *C.mantiqueirana* and C.odoratavar.botryoides.

Character	*Cuscutamantiqueirana*	Cuscutaodoratavar.botryoides
Stems	Yellow-orange, purple-tinged or purple	Yellow-orange
Pedicels	1.5–4 mm, obconical	1–3 mm, cylindrical
Bracts	1.1–2.5 mm long	2.2–3.2 mm long
Flower length	(4–) 5–7 mm	3.8–5 mm
Calyx	2.8–4 mm long, divided 2/3–4/5 to the base, tube 0.5–1.5 mm long, lobes 2–2.5 mm long, ovate-oblong, longer than wide to as long as wide, the two external ones overlapping, usually not carinate, not auriculate at base.	2.8–3.4 mm long, divided ca. 1/2–2/3 to the base, tube 1–2 mm, lobes ovate-round, 1–2 mm long, wider than long, the two external ones carinate, broadly overlapping, auriculate at base.
Corolla	(4–) 4.5–7 mm long, tube 2.1–4.6 mm long, lobes 1.8–2.5 mm long.	Corolla 3.5–4.5; tube 2–3.5 mm long, lobes 1–1.8 mm long.
Infrastaminal scales	3–5 mm long, bridged at 0.7–1.6 mm, fimbriae 50–90, 0.5–1.2 mm long, thin-filiform without papillae.	2–3.7 mm long, bridged at 1–1.5 mm, fimbriae 60–110, 0.3–0.6 mm, often with 1–2 distal papillae.
Stamens	Anthers 0.8–1.2 mm long, filaments 0.6–0.8 mm long.	Anthers 0.6–0.8 mm long, filaments 0.4–0.6 mm long.
Pollen	3-zonocolpate,19–29.5 μm long and 17–20 μm wide, subprolate to spheroidal or suboblate, tectum microreticulate to reticulate, lumina 0.9–3.3 μm in diameter.	(3–) 4–5 colpate, 19–26 μm long and 18–26 μm wide, sphaeroidal to subsphaeroidal, tectum perforatum; puncta 0.4–1(1.2) μm in diameter.
Ovary	Ovary apex on both sides of styles risen to form 4 lobes or projections with stomata, 0.4–0.6 mm long.	Apex without lobes or projections but a few stomata may be present.
Styles and stigmas	Styles 0.3–0.9 mm long, 0.25–0.5 mm thick, cylindrical or slightly subulate, sometimes also with stomata at their base; stigmas 0.35–0.5 mm long and 0.5–0.7 mm wide	Styles 0.8–3 mm long, 0.25–0.35 mm thick, cylindrical; stigmas 0.3–0.4 mm long and 0.3–0.5 mm wide.
Capsule	Stomatiferous lobes form a collar around the large interstylar aperture	Without a collar around the large interstylar aperture.
Distribution	Brazil: Minas Gerais, Rio de Janeiro and São Paulo.	Argentina: Misiones; Brazil: Paraná, Santa Catarina.
Elevation	800–2360 m	3–150 m
Ecology	Montane rain forest	Resting in a forest or near rivers

##### Geographical distribution and ecology.

The new species is apparently endemic to Serra da Mantiqueira in Southeastern Brazil (states of Minas Gerais, Rio de Janeiro and São Paulo), where it occurs at elevations between 800–2360 m. The climate is mesothermic, characterized by distinct dry and rainy seasons, with an average temperature that depends on the elevation, but generally exceeding 10 °C (ICMBio 2018). Serra da Mantiqueira is part of the Atlantic Forest Biome and the habitat of *C.mantiqueirana* consists of montane and upper montane cloud forests (Segadas-Vianna and Dau 1965; Ururahy et al. 1983; Veloso et al. 2012). During field work conducted in Itatiaia National Park and Monte Verde, we observed *C.mantiqueirana* at forest edges, margins of roads and clearings as well as parasitizing isolated woody plants in the forest (Fig. 4A, B). The most common host is *Fuchsiaregia* (Vell.) Munz (Onagraceae; Fig. 4E), which is the most widely distributed species of this genus in Brazil, occurring throughout the distribution range of *C.mantiqueirana* (Berry 1989). *Fuchsiaregia* is currently accepted to include three subspecies, *regia*, *serrae* P. Berry, and *reitzii* P. Berry (Berry 1989), and future field work will have to determine the frequency of occurrence on these subspecies. To a less extent, perhaps as secondary hosts, *C.mantiqueirana* parasitizes other herbaceous or woody plants: Asteraceae (*Baccharis* L., *Lepidaploa* (Cass.) Cass., *Mikania* Willd., including *Mikaniamicrantha* Kunth, and other unidentified Asteraceae), Euphorbiaceae (*Croton* L.), Fabaceae, Melastomatacaeae, Primulaceae (*Myrsinevenosa* A. DC.), Polygonaceae (unidentified), Rubiaceae (cf. *Spermacoce* L.), Solanaceae (*Solanum* L.), and Styracaceae.

##### Phenology.

Flowering in Nov-Dec and Feb-Aug, which may depend on the elevation. Very few herbarium specimens possess capsules and seeds which suggests that plants are preponderantly xenogamous and also reproducing vegetatively (Wright et al. 2012).

##### Etymology.

The specific epithet is a feminine adjective that comes from the name of the mountain range to which the species is apparently endemic. The word “Mantiqueira” is derived from Tupi-Guarani meaning “mountains that cry” alluding perhaps to the plethora of dripping water, streams and rivers that are present during the wet season with abundant rainfall (Mendes Júnior et al. 1991).

##### Vernacular names.

The common names used in the area are: Cipó-chumbo, fios-de-ovos, erva-de-passarinho (although also commonly used for other species of *Cuscuta* that occur in the region).

##### Provisional conservation status.

The GeoCAT rapid assessment tool (Bachman et al. 2011) assigned an Endangered (EN) conservation status based on an EOO of 33011.111 Km^2^ and a Vulnerable (VU) status based an AOO of 504.000 Km^2^. Further field research is necessary to investigate possible additional distributional records in the region, and assess other factors such as habitat threats to determine if this species is in need of conservation in any of the parts of the extensive Mantiqueira mountain range.

##### Additional specimens examined.

Brazil. Rio de Janeiro: Itatiaia, 13 Apr 1963, *E. Pereira & C. Pereira 7559* (HB); idem, Km 7 da estrada de Registro para Planalto, 17 Feb 1969, *G.F.J. Pabst 9306* (MBM, HB); Parque Nacional de Itatiaia, 2 Nov 1965, *G.G. Eiten & L.T. Eiten 6528* (SP); idem, 2 Apr 1960, *O.M. Barth 7144* (IOC, US); idem Pico das Agulhas Negras, 2159 m, 1 May 1977, *D.M. Vital s.n.* (UEC, BR, NY); idem, Estrada Nova Km 8, 25 Mar 1942, *A.C. Brade 17266* (RB); idem, 2200 m, 1 May 1977, *J. Vasconcellos-Neto* et al. *s.n.* (UEC); Estrada do Ponto Zero para as prateleiras, 22°22'12"S, 44°42'31"W, 2380 m, 11 Dec 2002, *R. Marquete* et al. *3437* (RB; DNA accession 1123); Resende, West side of Mt. Itaiaia, at km 9–10 km on road from “Registro” to the shelter house, “Abrigo Rebouças”, 2100 m, 7 Nov 1965, *G.G. Eiten & L.T. Eiten 6682* (K, SP, NY, MO, UBC, US); Estrada vicinal de acesso ao Pico das Agulhas Negras, 24 May 1996, *G.F. Árbocz 2750* et al. (SP, NY, UEC, WLU). **Minas Gerais**: Alto Caparaó, Parque Nacional do Caparaó, Trilha Tronqueira ao Pico da Bandeira, 13 Mar 2010, *J.M. Silva & J. Cordeiro 7543* (MBM); Camanducaia, Monte Verde; idem, 3 Jun 1992, *R. Simão-Bianchini 317* (SPF, WLU); idem, 12 Jan 2020, *R. Simão-Bianchini 2332* (SP); idem, 16 Mar 1976, *H.F. Leitão Filho* et al. *1815* (UEC, SPSF, UB, WLU) idem, Serra da Mantiqueira, 11 Dec 2001, *L.D. Meireles & R. Balinello 770* (UEC); idem, Pico do Selado, 1810 m, 17 May 2002, *L.S. Kinoshita* et al. *72* (UEC); Itamonte, Parque Nacional do Itatiaia, 20 Nov 2018, *S.S. Silva* et al. *Itati 02*, *04*, *05* (SP, WLU); idem, 21 Nov 2018, *S.S. Silva* et al. *Itati 08* (SP, WLU; DNA accession 2436); idem, Itamonte, Serra Fina, Sítio Pierre, 2100 m, 20 Jul 2005, *L.D. Meireles* et al. *1843* (SP, UEC); Idem, 4 Apr 1995, 2100 m, *I. Koch & L.S. Kinoshita 449* (UEC); **São Paulo**: Campos do Jordão, Instituto Kurihara, 8 Jun 1940, *G. Hashimoto 262* (SP); idem, Parque Estadual Campos do Jordão, 1760 m, 8 Feb 1980, *R.A.A. Barreto 48* (SPSF); idem, Praia São José dos Alpes, 8 Jun 1992, *E. Gianotti* et al. *26667* (UEC); idem, 2 Aug 1980, *A.A.B. Rubens 48* (RB) Pindamonhangaba, P.E. de Campos do Jordão, 12 Apr 1985, *M.J. Robim 277* (SPSF); idem, 12 Apr 1985, *C. Proença & M.F. Bean 496* (CEN, UB).

## Discussion

### Systematics of *Cuscutamantiqueirana*-related group of taxa

Cuscutaodoratavar.boliviana Engelm. Trans. Acad. Sci. St. Louis 1: 477. 1859.

= C.bolivianavar.paranensis Hunz., Revista Argent. Agron. 14: 142.1947.

The morphological distinctiveness of *C.mantiqueirana* allows its unequivocal recognition as a new species even though we could not obtain molecular data for some morphologically similar taxa – *C.rotundiflora* and C.odoratavar.botryoides. The available molecular results agree with the morphological patterns observed in section Subulatae, and the similarity of *C.mantiqueirana*, C.odoratavar.botryoides, and *C.rotundiflora* strongly suggests a phylogenetic proximity of these taxa in clade B (Fig. 1). Nevertheless, a complete picture of the evolutionary relationships and systematics of this clade will require molecular results for all the taxa involved.

As indicated, *C.mantiqueirana* is most similar morphologically to C.odoratavar.botryoides (Table 1), a taxon that was described by Engelmann (1859) from “Southern Brazil” based on a single specimen, *Lobb 49* (K, MO). Engelmann (1859) viewed this variety as “intermediate” between *C.odorata* and *C.chilensis*. Subsequently, Yuncker (1922, 1932) maintained this taxon as a variety of *C.odorata*, but compared it with *C.globiflora*. More recently, Hunziker (1947) described C.bolivianavar.paranensis Hunz. from Misiones (Argentina) and Paraná (southern Brazil), which he considered to be most similar to *C.boliviana* (var. boliviana) and *C.cristata*. These two varietal names have been accepted by modern floristic overviews (e.g., Zuloaga et al. 2008; Flora do Brasil 2020), but the taxa themselves have remained little known until now. After examining the types and few specimens available for C.odoratavar.botryoides and C.bolivianavar.paranensis, we consider these names synonymous. Variety *botryoides* has priority at this rank (“the original subdivisional epithet”, a rule that had already been in place in 1947 when it was described by Hunziker (Art 55; Camp et al. 1948). Although it could not be included in the molecular study, C.odoratavar.botryoides is likely not related to C.odoratavar.odorata because at least some of its pollen grains are 4–5-colpate and its infrastaminal scale fimbriae have only 1–2 distal papillae. Thus, C.odorata is most probably polyphyletic; var. odorata shares affinities with the species of clade A (comprised of *C.foetida*, *C.purpurata*, *C.chilensis*, etc., Fig. 1), while var. botryoides is a member of clade B. The taxonomic rank and evolutionary relationships of var. botryoides remain to be solved by a future study.

### Stomatiferous protuberances

Stomatiferous structures have been recently documented in many *Cuscuta* species (reviewed by Clayson et al. 2014); however, their presence at the apex of the ovary and base of styles in *C.mantiqueirana* is a novel feature. Stomatiferous (multicellular) protuberances (SPs) develop during anthesis on the haustorial stems of species in subgenus Grammica, as well as on the calyx and corolla of flowers in species from multiple clades of subgenera *Cuscuta* and *Grammica* (Clayson et al. 2014). When present on the calyx and corolla, SPs are diversely shaped (e.g., tubular, hemispherical, conical, crests) and have evolutionary and taxonomic significance (Costea and Stefanović 2010; Costea et al. 2011a, 2011b, 2013). SPs on the flowers are characteristic of species that have evolved in areas with a marked dry season (Clayson et al. 2014). The water loss through the SPs stimulates the hosts to absorb more water by increasing the negative pressure/tension in the xylem of the host, via the haustoria connection (Clayson et al. 2014). Some taxa of section Subulatae also have SPs on the calyx lobes; for example, the species epithets of *C.cristata* and *C.alatoloba* Yunck. refer to the presence of crests on the calyx lobes, but their authors (Engelmann 1859; Yuncker 1932) did not know that these structures bear stomata or their function. The discovery of stomatiferous structures on the ovary and style base of *C.mantiqueirana* improves the knowledge about the diversity of SPs, and strongly suggests that the morphology of floral SPs will play a significant role in the species-level systematics of section Subulatae.

### Specimens of C.odoratavar.botryoides examined

Argentina. Type of C.bolivianavar.paranensis; Missiones, Posadas, en la costa del río, 15 Jul 1945, *Bertoni 1502* (LIL); idem, 2 Aug 1945, *Burkhart 1626* (CORD). Brazil. Type of C.odoratavar.botryoides; “S. Brasil”, *Lobb 49* (K, MO); Paraná: Mun. Paranagua, Ipanema, 27 Aug 1969, 3–5 m, *Hatschbach 22109* (K, MBM, MO); paratype of C.bolivianavar.paranensis; idem, 25 Oct 1929, *Hoehne s.n.* (SP 24476).

## Supplementary Material

XML Treatment for Cuscuta mantiqueirana

## Appendix I

**Taxa, DNA accession numbers, sources of plant material from which DNA was extracted, and GenBank accession numbers for nrITS sequences used in this study. Extraction numbers following species names are indicated on the phylogenetic tree (Fig. 1). Abbreviations of herbaria in which the vouchers are deposited follow Index Herbariorum (Thiers 2018-continuously updated). In bold are indicated accession numbers for sequences newly generated for this study.**

Cuscutaaff.chilensis Ker Gawl.: **999**, *Hichins & Muñoz s.n.* (SGO), EF194525; **1000**, *Teiller* et al *2489* (SGO), EF194524; *C.chilensis*: **567**, *Ledingham 4455* (USAS), EF194520; **715**, *Arroyo* et al. *996099* (SGO), EF194521; **716**, *Morales and Cordoba s.n.* (SGO), EF194522; **967**, *Landrum 3392* (ASU), EF194523; **985**, *Muñoz and Meza 2202* (SGO), EF194519; *C.cockerellii* Yunck.: **1055**, *Straw 2267* (US), EF194518; *C.cristata* Engelm.: **939**, *Riggs 100* (F), EF194529; **1026**, *Landrum 3057* (ASU), EF194531; **1045**, *Hunziker 5047* (US), EF194530; *C.foetida* (var. foetida) Kunth: **496**, *Ollgaard & Balsev 8960* (F), EF194512; **922**, *Steyermark 53255* (F), EF194513; **1020**, *Sparre 16952* (TEX), EF194511; C.foetidaKunthvar.pycnantha (Benth.) Yunck.: **990**, *Lira 13* (SGO), EF194527; *C.friesii* Yunck.: **1076**, *Cabrera* et al. *21399* (LP), EF194536; *C.globiflora* Engelm.: **909**, *Vargas 684* (F), EF194533; **926**, *Buchtien 133* (F), EF194534; *C.grandiflora* Kunth: **540**, *Hutchinson & Wright 4305* (F), EF194535; *C.kilimanjari* Oliv.: **471**, *Knox 5020* (TRTE, IND), EF194528; *C.mantiqueirana* Costea, S.S. Silva, Sim.-Bianch., sp. nov.: **1123**, *Marquete 3437* (RB), MZ389691; 2436, *Silva* et al. *08* (SP/WLU), MZ389690; 2437, *Simão-Bianchini 1241* (SP/WLU), MZ389689; *C.microstyla* Engelm.: **707**, *Muñoz* et al. *3575* (SGO), EF194538; **987**, *Vargas & Farah 80* (SGO), EF194537; C.odorataRuiz & Pav.var.odorata: **912**, *Hutchinson 1055* (F), EF194514; **1024**, *Asplund 7737* (TEX/LL), EF194515; *C.paitana* Yunck.: **940**, *Haught 63* (F), EF194516; **941**, *Weberbauer 7762* (F), EF194517; *C.parodiana* Yunck.: **512**, *Krapovickas 37354* (F), EF194532; *C.purpurata* Phil.: **1432**, *Muñoz 5132* (SGO/WLU), MZ389688.
